# A Practical Update of Surgical Management of Merkel Cell Carcinoma of the Skin

**DOI:** 10.1155/2013/850797

**Published:** 2013-01-30

**Authors:** Patricia Tai

**Affiliations:** Division of Oncology, Allan Blair Cancer Center, 4101 Dewdney Avenue, Regina, SK, Canada S4T 7T1

## Abstract

The role of surgeons in the treatment of Merkel cell carcinoma (MCC) of the skin is reviewed, with respect to diagnosis and treatment. Most of the data in the literature are case reports. Surgery is the mainstay of treatment. A wide local excision, with sentinel node (SLN) biopsy, is the recommended treatment of choice. If SLN is involved, nodal dissection should be performed; unless patient is unfit, then regional radiotherapy can be given. Surgeons should always refer patients for assessment of the need for adjuvant treatments. Adjuvant radiotherapy is well tolerated and effective to minimize recurrence. Adjuvant chemotherapy may be considered for selected node-positive patients, as per National Comprehensive Cancer Network guideline. Data are insufficient to assess whether adjuvant chemotherapy improves survival. Recurrent disease should be treated by complete surgical resection if possible, followed by radiotherapy and possibly chemotherapy. Generally results of multimodality treatment for recurrent disease are better than lesser treatments. Future research should focus on newer chemotherapy and molecular targeted agents in the adjuvant setting and for gross disease.

## 1. Introduction

Merkel cell carcinoma (MCC) of the skin is also called trabecular carcinoma, cutaneous neuroendocrine carcinoma, or Merkeloma. It is an uncommon, highly malignant primary cutaneous carcinoma. It is thought to arise from Merkel's cells located in the basal layer of the epidermis and hair follicles, associated with sensory neurites in the dermal papillae, which are the mechanoreceptors in the skin.

MCC occurs mostly in white elderly with an equal incidence in men and women. About 78% of patients are older than 59 years. It may be slightly more common in females [1, 2]. There is no sexual predilection in the age group below 60 years. After 60 years, MCC are more often seen in female patients.

The tumor is most often located in the head and neck region (50.8%) or the extremities (33.7%). The exact etiology is not known, but is postulated to relate to sunlight, immunosuppression, and infection by a Polyoma virus. The discovery of Merkel cell polyomavirus is a major breakthrough [3]. MCC occurs more frequently in immunosuppressed patients, for example, transplant recipients or AIDS patients. The association of a polyoma virus with this tumor may explain the increased incidence in immunosuppression. The immunological aspect of the cancer is very interesting, with some case reports of complete or partial spontaneous remission [4].

The incidence rate is increasing which could be secondary to improved detection or an aging population. The incidence (per 100,000) of MCC in Finland in 1989–2008 was 0.11 for men and 0.12 for women, adjusted for age to the world standard population [5]. The Netherlands Cancer Registry from 1993 to 2007 recorded 808 MCC cases [6]: the annual age standardized incidence rate per 100,000 of MCC increased from 0.17 in 1993–1997 to 0.35 in 2003–2007.

Due to the rarity of the disease, most surgeons (except in specialized centers) do not have a lot of expertise with its management. There are some controversies in the literature regarding treatment options, especially in the adjuvant setting. The appropriate treatments in recurrent and metastatic disease become a dilemma due to its occurrence in the elderly who tend to tolerate aggressive treatment poorly.

Due to the previous reasons, this paper is written to assist the general and plastic surgeons. A systematic literature search was carried out using the term “Merkel cell carcinoma and surgery” on PubMed up to 24 June 2012 and summarized so it can be applied in the clinic easily. This paper will discuss the role of surgeons in

1. diagnosis,
2. biopsy and pathology evaluations,
3. pretreatment evaluations,
4. definitive surgery—sentinel lymph node (SLN) biopsy and nodal dissection,
5. referral for other treatments,
6. follow-up,
7. management of recurrence.

This paper concludes with a summary of the most up-to-date treatment algorithms, treatment outcomes in large series of MCC, and direction for future research.

## 2. Diagnosis of MCC

 In the clinic, only a presumptive diagnosis of MCC can be entertained, with more common skin cancers in the differential including basal cell carcinoma, squamous cell carcinoma, melanoma, and Kaposi sarcoma and lymphoma. It usually presents as a rapidly growing, painless, firm, nontender, shiny, bluish-red, intracutaneous nodule, generally of 0.5–5 cm (average 2.9 cm) in diameter. Heath et al. proposed the acronym AEIOU to describe the most common clinical characteristics: asymptomatic, expanding rapidly, immune suppression, older than 50 years, and UV-exposed site on fair skin [25].

The surgeon should be cautious that *atypical cases* can occur: transplant patients are affected at a younger age, with 29 percent being less than 50 years old compared to only 5 percent of the general population [26]. Sometimes it can take the form of a plaque. The tumor may appear in areas not exposed to sun, for example, extremities, trunk, genitalia, and perianal region in a random distribution. In addition, mucosal MCC had been reported, for example, nasal mucosa and nasopharynx [27]. Occasionally 12% (38/321) of patients can present as nodal mass without any obvious primary in the skin [28, 29].

## 3. Biopsy and Pathology Evaluations

Table 1 shows the summary for pathological diagnosis of MCC. Fine needle aspiration cytology (FNAC) enables an early noninvasive diagnosis of this aggressive tumor to facilitate early planning of surgery. FNAC can be easily performed in elderly patients compared to excisional biopsy. The cytological features in aspirate (stained with Pappenheim and Papanicolaou staining) include increased cellularity, noncohesive groups of small-to-medium size malignant cells with uniform, round-to-oval nuclei with moulding effect, fine chromatin, multiple micronucleoli, and scanty cytoplasm [30]. Since this tumor has a highly malignant potential for local recurrence, nodal and distant spread, and very often is combined with other tumors, *it is important to perform FNAC or biopsy of different lesions in the same patient. Collision tumors* have been reported in which MCC coexists with other skin malignancies [31].

 Pathologists should follow standard protocol for the examination of MCC specimens [32]. Definitive diagnosis is made by histological, especially immunohistochemical methods (detection of intermediate filaments and neuroendocrine markers) [2]. 

 Histologically, MCC arises in dermis and extends into the subcutis. The epidermis is infrequently involved, and the overlying skin is rarely ulcerated. The tumor can consist of isolated cells, loose cohesive sheets, and rosette-like structures.

The 3 main histologic patterns are (1) solid type—most common type, composed of irregular groups of tumor cells interconnected by strands of connective tissue, (2) trabecular type—well-defined cords of cells that form invading columns or cords, and (3) diffuse type—exhibits poor cohesion and a lymphoma-like diffuse type of growth.

 Neoplastic cells in MCC are round to ovoid and very uniform. Finely granular chromatin and frequent mitotic figures are observed. Under close scrutiny small faintly stained juxtanuclear “caps” were seen. The paranuclear globular coexpression of cytokeratin and neurofilaments by an undifferentiated dermal tumor is of significant help in diagnosing MCC and differentiating it from small-cell carcinoma [33]. The electron microscopy demonstrates the pathognomonic features for this tumour: dense-core neurosecretory granules with diameter of 100–250 nm surrounded by whorls of intermediate filaments [34]. Under the scanning electron microscope, numerous finger-like processes, ranging from 0.1–0.25 micron in diameter and 2.5 micron in length, had been described by Yamashita et al. [35]. 

 Immunocytochemical results are universally positive for cytokeratin [36]. Antibodies associated with epithelial derivation include antikeratin monoclonal antibody AE1/AE3, polyclonal anti-keratin, and monoclonal anticytokeratin cocktail (MAK-6), as well as a monoclonal antibody against epithelial membrane antigen (EMA). Positive keratin labeling (AE1/AE3, MAK-6) of filaments arranged in paranuclear aggregates, with presence of cytoplasmic synaptophysin helps to make the diagnosis [37]. Neuron-specific enolase (NSE) positivity is diffuse, although a weak dot-like positivity is seen in some cells. Leukocyte common antigen is universally negative. There could be conflicting immunostaining results. An example of positivity for chromogranin in the primary tumor but negative in the cytologic material was described by Gupta and Teague [38]. Other positive markers include Ber-EP4, an immunohistochemical marker used to identify carcinoma. Thyroid transcription factor-1 had been reported to be positive in MCC [39], *so it is unreliable by itself to differentiate from metastastic small-cell carcinoma of the lung metastasizing to the skin.*

Recent studies demonstrate chromosomal abnormalities in chromosomes 1, 11, and 13 [40, 41]. Comparative genomic hybridization analysis revealed a pattern of gains and losses that closely resembles that seen in small-cell lung cancer. Losses were seen for chromosomes 3p (46%), 5q (21%), 8p (21%), 10 (33%), 11q (17%), 13q (33%), and 17p (25%). Significant gains were seen for chromosomes 1 (63%), 3q (33%), 5p (38%), 8q (38%), 19 (63%), and X (41%), with smaller numbers having gains for chromosomes 6, 7, 20, and 21 [42].

 Diagnostic pitfalls include the following.

1. Coexistence of primary cutaneous MCC in association with squamous and basal-cell carcinoma [43]. This has the implication that an ordinary squamous or basal cell carcinoma should be sectioned thoroughly to avoid missing the aggressive MCC component.
2. Presence of desmoplasia may mask the diagnosis of MCC [44].
3. Malignant lymphoma is an important differential diagnosis of cutaneous small round blue cell tumors. Immunohistochemistry and, if necessary, polymerase chain reaction and sequencing are useful tools to differentiate them [45].

The Johns Hopkins School of Medicine applied molecular techniques to detect Merkel cell virus (MCV) in sentinel lymph node (SLN) [46]. Eight out of 25 (32%) samples had detectable MCV without microscopic disease. This may identify a subset of patients who would benefit from adjuvant nodal treatment.

## 4. Pretreatment Evaluation

### 4.1. TNM Staging System

Table 2 shows the 2010 tumor (T) node (N) metastasis (M) staging system that is supported by both the American Joint Committee on Cancer (AJCC) and the International Union for Cancer Control (UICC) [23, 47]. This staging system was based upon an analysis of 5823 prospectively enrolled MCC cases in the National Cancer Database registry [7]. In this database, the 5-year overall survival was 40%, and relative survival (compared with age- and sex-matched population data) was 54% (Table 3). For cases presenting with local disease only, smaller tumor size was associated with better survival (stage I, ≤2 cm, 66% relative survival at 5 years; stage II, >2 cm, 51%; *P* < 0.0001). About 1/3 of patients with clinical negative regional lymph nodes had microscopic involvement upon pathologic examination. The 2010 TNM staging system has been criticized for its complexity. Its merits are the following: (1) it is more in line with staging system of other skin cancers. (2) The distinction between pN0 versus cN0 is important: 5-year overall survival is 76% versus 59%, *P* < 0.0001. Readers should note that most of the older literature used simpler staging systems.

### 4.2. Staging Workup

Table 4 summarizes the staging work up. Computerized tomography (CT) scans had low sensitivity (20%) for nodal disease and low specificity for distant disease (only 4 of 21 “positive” scans were confirmed during 6 months of followup) [48]. Positron emission tomography (PET) is a useful staging technique. It makes the differential diagnosis of the lymph node involvement from MCC and chronic lymphocytic leukemia (CLL) or benign conditions possible [49].

 The use of octreotide scan is still considered investigational.

### 4.3. Prognostic Factors

Prognostic factors have been used as the basis for treatment recommendation. Postoperative radiation or chemotherapy may reduce the risk of recurrences, and it should be considered for patients with poor prognostic features. There is no correlation between the detection of the Merkel cell polyomavirus in the primary tumor and the appearance of metastases [50].  Table 5 summarizes the prognostic factors. Generally prognostic factors can be divided into host and tumor factors as the following.

#### 4.3.1. Host Factors

MCC in buttock/thigh area or trunk has the worst prognosis, and that on the distal extremities has the best prognosis [8]. Head and neck disease has the highest risk of local recurrence, which occurred in 62.5% of this group. Among the 2104 patients with head and neck MCC in the Surveillance Epidemiology and End Results (SEER) database of the United States, scalp tumors are significantly larger (10.4% >5 cm, *P* = 0.0001) and more likely to present with distant metastasis (8.7%, *P* = 0.07) than other head and neck tumors [51]. The most likely reason is late detection of scalp tumors as compared to other sites. Lip tumors have the highest rate of invasion into bone, cartilage, and muscle (13.7%, *P* = 0.012), and ear tumors have the highest rate of nodal metastasis (63.2%, *P* = 0.011). 

Males are reported to have a worse prognosis independent of stage and extent of therapy: 3-year overall survival of 35.6% for men and 67.6% for women [5, 52]. 

#### 4.3.2. Tumor Factors

Tumor stage is a very important prognostic factor. Even for stage I, patients have a high risk of disease progression. In the classical series of Savage, 53% of patients with stage I develop regional lymphadenopathy or visceral metastases. The median survival for all disease stages was 47 months [53]. Similarly, another study noted a high rate of nodal recurrence among patients with stage I disease who had undergone treatment of the primary site only. These patients may have benefited from sentinel lymph node biopsy (SLNB) and subsequent treatment of the nodal basin if micrometastatic disease was present, as the number of involved nodes impacted negatively on overall survival [54]. 

 Despite critiques of the current staging system being too complicated for use, it does have merit to distinguish between clinical versus pathological nodal involvement. The presence of clinically positive lymph nodes was found to be associated with increased disease-specific death according to a large prospective database from Memorial Sloan Kettering Cancer Center [21]. Large tumor size, small-cell size, and high mitotic rate are associated with a low survival rate. When cell size was excluded, male sex and depth of invasion were associated with a worse survival, although these were not statistically significant [55]. The 5-year distant-recurrence crude cumulative incidence (CCI) was higher in clinically node-positive patients compared with node-negative patients (37% versus 12%; *P* = 0.005) [56]. Patients with occult primary had a 5-year distant recurrence CCI of 49% (*P* = 0.023). Among node-positive patients, those with ≤2 versus >2 metastatic lymph nodes, the 5-year regional-recurrence CCI was 0% versus 39% (*P* = 0.004) when treated with surgery alone. This is an interesting observation as the cut-offs for poor prognosis for prostate and breast cancers are also two involved nodes [57, 58]. 

Three cell proliferation markers were thought to be useful in predicting the aggressiveness of MCC: (1) p53, a tumor suppressor protein, (2) Ki-67, a marker of cell cycling, and (3) proliferating cell nuclear antigen (PCNA). Carson et al. [59] reported on 20 MCC patients. PCNA was positive in only 1 patient, while Ki-67 was positive in all patients, making these 2 markers unsuitable for predicting recurrence.

A recently discovered prognostic factor is peptidyl-prolyl *cis*-transisomerase (PIN1) [60]. An overexpression might cause cell cycle arrest and growth inhibition by binding to the p53 protein, a process leading to p53 stabilization. A retrospective analysis of 27 MCCs showed a significantly better overall survival in patients with an overexpression of PIN1 than in patients with a weak PIN1 expression (*P* = 0.031), but expression was not significant for disease-free survival (*P* = 0.821). The 5-year overall survival rate was 14.4% in patients with weak and 50.9% in patients with overexpression of PIN1. Further studies should be done to confirm this finding.

Tenascin-C (Tn-C), a large extracellular matrix glycoprotein, is associated with invasion and cellular proliferation. It had been shown to correlate with prognosis in different tumors [61]. Immunohistochemistry can be used to investigate the expression of Tn-C in MCC specimens. Its expression correlates significantly with large tumor size. It is also frequently expressed in primary tumors with metastatic dissemination and in tumors with high proliferative indices. Further clinical studies should validate these markers and try to discover new markers, for example, oncotype or chromosomal mutation.

## 5. Definitive Surgery

### 5.1. Resection Margins

Table 6 summarizes basic surgical principles for MCC [9]. Achieving negative margins with the initial resection appears to be important for long-term disease control. In a review of 661 published cases by Tai et al. [8], complete excision was associated with a statistically significant improvement in overall survival and a trend toward better disease-free survival (*P* = 0.08).

 Wide excision of the primary MCC tumor is the standard approach to the initial management of the primary tumor. Shaw and Rumball [62] in 1991 suggested minimal treatment should consist of wide surgical excision of the primary with a margin of 2.5–3 cm. Now the general recommendation is a margin of at least 1 to 2 cm. A 3 cm margin is not required provided postoperative radiotherapy is used [63]. 

### 5.2. Mohs Micrographic Surgery

Because MCC often has extensive vertical growth and sometimes extends into muscle, adequate deep margin is also vital. Mohs micrographic surgery has been advocated to improve local tumor control compared with standard wide excision. With this approach, 100% of all major borders, including the deep margins, are evaluated histologically. It offers a superior alternative to standard excision [64], with increased cure rates and tissue conservation. For facial MCC, removal by Mohs surgery followed by radiation is less disfiguring.

Mohs micrographic surgery is cost effective in the American health care system because billing for the surgeon-pathologist and laboratory processing is bundled together [65]. However, Mohs micrographic surgery may be more expensive in European systems because the surgeon, pathologist, and laboratory may bill separately.

There is a controversy of the necessity of adjuvant RT after Mohs surgery. In Boyer et al.'s study of 45 patients with stage I MCC who were histologically and clinically free of disease after Mohs excision, the value of adjuvant RT after Mohs surgery was evaluated [66]. Twenty patients received elective postoperative radiation to the primary site, and 25 patients had no adjuvant radiation therapy. One marginal recurrence (4%) and 3 in-transit metastases were observed in the Mohs surgery alone group, whereas none were observed in the Mohs surgery and radiation group. The proportion of patients with these events was not significantly different between treatment groups. According to the authors, adjuvant radiation appears unessential for local control of primary MCC lesions completely excised with Mohs micrographic surgery. 

 The experience of the 13 patients treated with Mohs surgery by O'Connor et al. is similar [67]. Mean followup for the group treated with Mohs surgery was 36 months. Only one of 12 (8.3%) Mohs-treated patients with histologically confirmed clearance had local persistence of disease. This patient underwent a second Mohs excision and remained disease-free for 84 months. Regional metastasis developed in four of 12 cases (33.3%). Regional metastasis developed in none of the four patients treated with radiotherapy after Mohs surgery and in four of eight patients treated with Mohs surgery without postoperative radiotherapy. 

In Snow et al.'s series, local control of the primary MCC was achieved in 70% of patients (7 of 10) using combined Mohs excision and radiation [27]. Two recurrent cases had primary tumors larger than 3.5 cm in diameter, while the other case was unresectable by Mohs surgery. Tumor size appeared to determine the degree of local control. When the postoperative Mohs defect was less than 3.0 cm in diameter, local and regional control appeared to be more favorable. 

To conclude, the addition of RT after Mohs surgery may have a small though statistically nonsignificant benefit to reduce local/regional recurrence. For large MCC or those with high-risk features, adjuvant RT is definitely indicated. It is also recommended for patients who are unable to have complete excision or if complete histologic margin control is unavailable and should be considered for patients with large or recurrent tumors.

### 5.3. Surgical Management of Nodal Region

The other important progress in recent years is in the surgical management of nodal region. Regional lymph node metastasis occurs early and frequently, with a 79% overall incidence observed during the course of the disease [68]. Therefore, patients with clinical and radiographical negative lymph node status should routinely undergo sentinel lymph node biopsy (SLNB). SLNB is important for both prognosis and therapy [48]. 

A double-indicator technique with technetium-99 sulfur colloid and isosulfan blue is used [69, 70]. Preoperative lymphoscintigraphy may be done the night before so that the surgery can be scheduled as the first case of the following day. The surgeon should master the techniques of preoperative lymphoscintigraphy and intraoperative lymphatic mapping. An SLN is defined as a blue, “hot,” and any subsequent lymph node greater than 10% of the ex vivo count of the hottest lymph node [71]. Any enlarged or indurated lymph node in the nodal basin should be excised. Every surgeon who uses blue dye should be aware of the potential adverse reaction to isosulfan blue and treatment for such a potential fatal reaction. A complete lymph node dissection should be done if the SLN is found to be positive. In a study from the Institute Gustave Roussy, identification of microscopic nodal metastases would be followed by complete lymph node dissection and adjuvant radiation therapy to the lymph node basin [72]. If expertise for SLNB if not available, patients should have elective dissection or radiation therapy. 

Occult metastases were observed in 36% of SLNB in MCC [10]. False-negative lymph nodes were found in 30% of the patients. Immunohistochemical reevaluation decreased this figure to 22%. Therefore for MCC, false-negative sentinel lymph nodes should be avoided by using immunohistochemistry. Examination of hematoxylin and eosin (H&E) sections alone is insufficient for excluding micrometastatic MCC [73]. Su et al. observed the greatest sensitivity and specificity with anti-CK-20 antibody in identifying micrometastatic MCC in sentinel lymph nodes [74]. Another recent progress to minimize the chance of a false-negative SLNB is intraoperative imprint cytology (IIC). It can potentially avoid second operations for completion lymphadenectomy when nodal metastases are found during nodal staging with SLNB. Wong et al. performed successful SLN mapping procedures on 18 patients. IIC was negative in 84.2% (16) cases. Three false-negatives occurred with IIC, but there were no false-positives, making the sensitivity 33 percent and the specificity 100 percent. [69]

At one time, it was questioned if small MCC ≤1 cm needs SLNB. Stokes et al. reported that MCC 1 cm or smaller are unlikely to harbor nodal metastases [75]. Only two patients (4%) with tumors ≤1.0 cm had regional lymph node metastasis, compared with 51 (24%) of 213 patients with tumors more than 1.0 cm (*P* < 0.0001). With tumor size 1 cm or less, 2/54 patients (4%) had clinical regional node metastases at diagnosis; none of the remaining 52 patients with tumor size 1 cm or less and clinically negative nodes were found to have pathological nodes on surgical staging at the time of presentation. In the study of Allen et al. [76], out of 26 patients in which SNB was performed, 5 had nodal metastases, and out of these 1 had a tumor size of 1 cm. In his follow-up paper in 2005 [77], operative nodal staging was performed in 71 patients with clinically negative nodes, and a total of 16 patients (23%) had positive nodes. Positive nodes were discovered in 24% of patients with tumors <2 cm in diameter and in 20% of patients with tumors 2 cm or more in diameter (*P* = 0.71) [77]. In another study, Tai et al. [19] combined the data of 145 Canadian and French cases with 288 cases in the literature. Among them, 105 patients had primary tumor size ≤1 cm. Nodal metastases occurred clinically at presentation in 9/105 (9%) patients, and they concluded that the rate of nodal metastases is too high to obviate sentinel node biopsies even for these small tumors. Other authors also agree that routine omission of sentinel lymph node biopsy for MCC ≤1 cm is not justified [78].

As expected, factors associated with SLNB positivity are primary tumor size (25% ≤2 cm versus 45% >2 cm; *P* = 0.02) and presence of lymphovascular invasion (LVI) (55% LVI positive versus 4% LVI negative; *P* < 0.01) [11]. Increasing clinical size, tumor thickness, mitotic rate, and infiltrative tumor growth pattern were significantly associated with a positive SLN [79]. By using the growth pattern and tumor thickness model, no subgroup of patients was predicted to have a lower than 15% to 20% likelihood of a positive SLN. This suggests that all patients presenting with MCC without clinical evidence of regional lymph node disease should be considered for SLNB.

Outcomes of patients with and without positive SLN are very different. From a series of Mayo clinic, 40 of 60 patients (67%) had a biopsy-negative SLN; 97% of this group had no recurrence at 7.3 months median followup [80]. Twenty patients (33%) had a biopsy-positive SLN; 33% of this group developed local, regional, or systemic recurrence at 12 months median followup. Risk of recurrence or metastasis was 19-fold greater in biopsy-positive patients (odds ratio, 18.9; *P* = 0.005). None of 15 biopsy-positive patients who underwent therapeutic lymph node dissection experienced a regional recurrence; 3 of 4 who did not receive therapeutic lymphadenectomy experienced regional recurrence. Therefore, patients with SLNB positive status must undergo either complete lymph node dissection (CLND) or adjuvant nodal radiotherapy. Complete lymph node dissection is currently considered the first line treatment for micrometastasis, but nodal radiotherapy has been used with success when there is a high morbidity associated with CLND [81, 82]. In an Australian study of 11 patients [83], 3 had a positive SLNB, and, despite adjuvant radiotherapy, 2 of these 3 developed regional lymph node recurrence. Of the remaining 8 patients who had a negative SN biopsy, however, 5 also had regional nodal recurrences. There were 9 patients who received adjuvant radiotherapy to the primary site, with no in-field recurrences and 8 who received radiotherapy to their regional nodal field, with only 2 developing regional nodal recurrences—both were SLNB positive. 

In conclusion, the use of the SNLB for MCC will reduce the number of unnecessary lymphadenectomies, enable identification of microscopic metastases to lymph nodes, and improve the stratification and accrual of patients into adjuvant treatment protocols. It limits the potentially unnecessary morbidity of more comprehensive lymph node dissections in MCC patients who do not yet have metastatic involvement. The occurrence of MCC in a relatively older population makes comorbid conditions a key factor in treatment planning.  Table 7 shows a summary of important series of SLNB. 

### 5.4. Surgical Management of Different Sites

#### 5.4.1. Head and Neck

The overall survival of head and neck MCC at 5 years postoperatively is between 40% and 68% [84]. The head and neck region has a notoriously variable lymphatic drainage pattern [85]. Consequently, the incidence of successful SLNB in the head and neck is considerably lower compared to the SLNB on the trunk and extremities. Occasionally, head and neck lymphoscintigrams fail to identify a definitive lymphatic drainage pattern, making preoperative and intraoperative identification of sentinel nodes very difficult. A retrospective study was performed on head and neck melanoma and MCC patients from August 1997 to August 2002 [86]. Technetium-99m sulfur colloid was the radioactive tracer used by the nuclear medicine department to perform the lymphoscintigrams. Of the 74 patients who underwent preoperative lymphoscintigraphy in preparation for performing a SLNB, 5 (6.8%) were found to have nonlocalization of a sentinel node(s). Two of the 5 patients underwent reinjection of the radioisotope but still failed to further localize the radiotracer. All 5 patients went on to have wide local excision of the primary cancer on the day of the lymphoscintigram, as well as undergoing intraoperative examination of all head and neck nodal basins with a handheld gamma detector. No focal areas of radiation were identified and no lymph nodes were biopsied. The nonlocalization rate of 6.8% was clinically important and reflects either the inherent difficulty in imaging the head and neck region and/or the possible rapid rate of dye washout via multiple lymphatic drainage pathways that exist in this location.

In another study from University of Miami [87], lymphatic drainage to areas outside of the expected lymphatic basins occurred in 13.6%. Approximately 7% of tumors on the head and neck drain to contralateral SLN [88].

Surgical complications were rare [82]. No temporary or permanent dysfunction of facial or spinal accessory nerves occurred with sentinel node biopsy. Schmalbach et al. also concluded that SLNB is safe and reliable for regional staging of MCC of the head and neck [89]. They also found anti-CK-20 antibody to be crucial in identifying micrometastatic MCC in SLN when hematoxylin-eosin-stained sections were negative.

Useful examples of surgical techniques can be found in the literature. In the retrospective study of 22 patients with head and neck MCC from Mayo clinic [90], all patients who underwent wide local excision of the primary tumor with dissection of the lymphatic drainage basin were alive at 2 years as opposed to 68% who had wide local excision alone and 33% who had Mohs surgery. Therefore, wide local excision and dissection of the lymphatic drainage basin provided the best overall survival. 

Two subsites in the head and neck region are particularly interesting: the eyelid and the ear. MCC of the eyelid tends to present as a bulging painless erythematous nodule, with overlying telangiectatic blood vessels near the lid margin of elderly patients. The diagnosis can be difficult as it can be misdiagnosed as lymphoma, oat cell carcinoma, malignant melanoma, sweat gland tumors, or benign conditions like chalazion. It frequently invades lymphatic vessels. One-third of the tumors recur, and there is a high rate of metastasis. The estimated 5-year survival rate is 38% [91]. Wide surgical resection and reconstructive procedures should be followed by routine postoperative irradiation. Tumor resections are recommended to include a 2-3 cm tumor-free margin around the primary lesion when possible, but this is often difficult to achieve, where Mohs micrographic surgery has proved to be effective. In an American series of 14 cases of eyelid MCC, only 2 patients (14%) received prophylactic therapy beyond wide surgical excision [92]. Three patients (21%) had recurrences, none of whom initially received adjuvant therapy (i.e., radiation therapy, lymph node dissection, and/or chemotherapy) after wide surgical excision. Coskuncan et al. reported a patient who was treated with wide resection and the Cutler-Beard technique and then scheduled for radiotherapy [93]. In another study, an MCC of the upper lid was resected and the defect closed by a temporal skin flap and a temporary cantholysis [94].

Different treatment options for MCC in the ear are available [95]. Surgical options include superficial or total parotidectomy with facial nerve preservation, SLNB, followed by modified radical neck dissection in case of nodal involvement and/or elective neck radiotherapy. Some favor immediate reconstruction and would use a lower trapezius island flap or a large rotational flap, while others prefer primary closure or skin graft. Some prefer combined therapy, giving radiotherapy to the primary area and the neck postoperatively at a dose of 55–60 Gy (Gray), while others would treat the primary site postoperatively with 59.4 Gy and the neck primarily with radiotherapy with 50.4 Gy.

#### 5.4.2. Trunk

In the trunk, prediction of the lymphatic drainage by applying conventional anatomical knowledge from the lymphatic atlas is not easy. Choe et al. described an MCC lesion situated in the midline on the back and at the border zone in the lumbar area from which it could drain superiorly to the axillary lymph node(s), inferiorly to the inguinal node(s), or to both directions [96]. Lymphoscintigraphy was very helpful in this situation.

#### 5.4.3. Extremity

For extremity MCC, en bloc primary and nodal treatment is not possible. SLNB should be recommended [97]. Patients must be referred postoperatively for adjuvant radiotherapy.

## 6. Referral for Other Treatments

 The histological similarities with small-cell carcinoma have prompted physicians to adopt treatment approaches along the same line as small-cell carcinoma, that is, chemotherapy and radiotherapy. All MCC patients should be managed by a multidisciplinary team. Surgeons should always discuss and/or refer to the oncologists. 

### 6.1. Adjuvant Treatment

Adjuvant radiotherapy is definitely recommended to the resection site for lesions thought to be at increased risk of local recurrence: close or positive margin, tumor ulceration and deep invasion, large tumor size, and lymphovascular invasion. Radiotherapy doses range from 40 Gy/15 fractions/3 weeks to 50 Gy/25 fractions/5 weeks in the literature. Shorter regimens could be considered in patients too ill for prolonged radical treatment. Practical management of these often elderly patients can be difficult; one has to evaluate the inconvenience of fractionated radiotherapy in prophylactic nodal irradiation against the objective of avoiding extensive surgery as initial treatment or on relapse. 

There are numerous retrospective studies favoring the use of adjuvant radiotherapy [98–101]. The importance of postoperative radiation therapy in the treatment of MCC is illustrated by the series from Meeuwissen [102]. All of the 38 patients treated with surgery alone relapsed with a median time to recurrence of 5.5 months, while 10 of the 34 patients treated with surgery plus RT relapsed with a median time to recurrence of 16.5 months. Similarly, Kukko et al. noticed that none of the patients with stages I-II disease who had received postoperative radiotherapy to the tumor bed had a local recurrence [5].

Another retrospective study from Switzerland on 180 patients treated between February 1988 and September 2009 compared patients who had surgery alone with patients who received surgery and postoperative RT or radical RT [22]. It is noteworthy that the median followup was 5 years (range, 0.2–16.5 years) and the majority of patients had localized disease (*n* = 146), and the remaining patients had regional lymph node metastasis (*n* = 34). Local relapse-free survival (LRFS), regional relapse-free survival (RRFS), distant metastasis-free survival (DMFS), and disease-free survival (DFS) rates were improved by adjuvant radiotherapy and remained significant with multivariable Cox regression analysis. However overall survival (OS) was not significantly improved by postoperative radiotherapy (56% versus 46%; *P* = 0.2).

 A very large database from Surveillance Epidemiology and End Results (SEER) registry with 1,665 cases of MCC also supports adjuvant radiotherapy [18]. Adjuvant radiotherapy was a component of therapy in 40% of the surgical cases. The median survival for those patients receiving adjuvant radiotherapy was 63 months compared with 45 months for those treated without adjuvant radiotherapy. The use of radiotherapy was associated with an improved survival for patients with all sizes of tumors, but the improvement with radiotherapy use was particularly prominent when analyzing those patients with primary lesions larger than 2 cm. There is a significantly decreased incidence of local and regional recurrences when adjuvant radiotherapy is used. These observational results include many patients in whom the initial resection may have been incomplete or inadequate, and the precise role of adjuvant radiotherapy is less certain with a wide resection and negative surgical margins.

 When adjuvant radiotherapy is given, most radiation oncologists recommend treatment of both the surgical bed and the draining regional lymphatics to avoid geographic miss, if technically feasible. For MCC in the lower limb or buttock, the addition of radiotherapy improved relapse-free survival (RFS) (*P* < 0.001), as did radiotherapy to the inguinal nodes (*P* = 0.01) or primary site and inguinal nodes (*P* = 0.003) [103]. Hence elective treatment should be given to the inguinal nodes to reduce the risk of relapse. 

 Oncologists may question that if MCC is treated like small-cell lung cancer, would there be any role for prophylactic cranial irradiation? MCC rarely involves the brain, with only 33 such reported cases as at 2011 [104]. In addition, prophylactic cranial irradiation can result in cognitive impairment so it is not recommended as standard of care in MCC. 

 Summary of common indications for adjuvant radiotherapy is shown in Table 8. Since many studies have demonstrated lower recurrence rate with adjuvant radiotherapy, surgeons should always refer patients to the radiation oncologists for an assessment. Unless a very wide local excision has been performed, patient will likely benefit from adjuvant radiotherapy.

The role of adjuvant chemotherapy is very controversial. The 5-year disease-specific survival of patients was about 60% without and 40% with adjuvant chemotherapy [105]. However, likely cases with poor risk features will be selected for adjuvant chemotherapy. The lack of benefit could be related to cases entered into phase II or III studies that may not have very high risk. Other issues call into question the routine use of adjuvant chemotherapy in MCC:

1. morbidity and mortality,
2. rapid development of resistance of gross disease to chemotherapy,
3. chemotherapy suppresses immune function that may play a large role in defending the host from the development and progression of MCC [106].

Veness did not recommend adjuvant chemotherapy for node-negative patients and only selectively to node-positive patients at high risk for systemic failure (i.e., multiple positive nodes and extranodal extension) [105]. Adjuvant chemotherapy may be considered for selected node-positive patients, as per National Comprehensive Cancer Network (NCCN) guideline [13].

### 6.2. Inoperable or Unresectable Cases: Radiotherapy as Alterative Treatment

The options are RT, chemotherapy, octreotide, and hyperthermia treatment. 

Radiotherapy is useful as monotherapy in patients with inoperable disease because of age or location and was found to produce similar outcomes to conventional therapy of surgery with adjuvant radiotherapy [107]. Traditionally, involved nodes have been managed with resection but, currently, there are protocols exploring the use of concurrent chemoradiation as definitive treatment. Fang et al. reported a cohort of 50 patients with node-positive MCC. Outcome of patients given lymph node irradiation as definitive therapy was compared with patients treated by completion lymphadenectomy (CLND) [108]. Among them, 26 patients presented with microscopic lymph node disease and 24 patients with palpable lymph node involvement. Regional control for patients with microscopically involved lymph nodes was 100% regardless of treatment modality-definitive lymph node irradiation (*n* = 19) or CLND ± radiotherapy (*n* = 7) with median followup of 18 months. Patients with clinically positive lymph nodes had 2-year regional recurrence-free survival rate of 78% and 73% in the definitive lymph node irradiation (*n* = 9) and CLND ± radiotherapy (*n* = 15) groups, respectively (*P* = 0.8) with a median followup of 16 months. This is the largest series published to date of radiation monotherapy as regional treatment for lymph node-positive MCC. There was no difference in overall survival. In another study, Cotlar et al. reported on eight patients, and one patient had recurrence within an irradiated field [109]. As to date, surgery is still the standard of care for MCC, unless the patient is inoperable or the lesion is unresectable.

Chemotherapy can be used as

1. neoadjuvant treatment prior to surgery to allow shrinkage [110],
2. concurrent chemoradiation as in small-cell lung cancer definitive treatment,
3. palliatively for locally advanced disease or metastastic disease.

Even for patients with relatively poor general condition, oral etoposide can be given with durable response [111]. 

There are other options for systemic treatment, for example, tumor necrosis factor [112] and interferon [113]. Newer molecular targeted agents, for example, imatinib and pazopanib, [114, 115] have promising preliminary results. Receptors for somatostatin have been found in MCC and demonstrated in vivo by octreoscan. Octreotide, the most important somatostatin analogue, is often used in neuroendocrine tumor management. It has a favorable toxicity profile. There is a case report of a patient suffering from metastatic MCC; because he was elderly, neither chemotherapy nor radiotherapy was possible [116]. The presence of receptors for somatostatin analogues (octreoscan) allowed treatment with octreotide causing the immediate disappearance of metastasis. After ten months of treatment the patient achieved complete remission. 

Lastly, hyperthermia also appeared to be beneficial [117]. More studies are needed before it can be used routinely for MCC patients. 

## 7. Follow-Up

Patients with MCC requires continual followup due to the high incidence of local, regional, and disseminated recurrences. Generally, 80% of the recurrences occur within 2 years and 96% within 5 years [56]. The frequency of followup should be individualized according to risk factors as discussed previously and the available therapeutic options. For those with salvage options available, the followup should be vigilant. According to National Comprehensive Cancer Network (NCCN) guidelines [9], physical exam including complete skin and lymph node examination should be performed every 3–6 months for 2 years and every 6 to 12 months thereafter. This is justified by the frequency of skin and nodal recurrence and the lack of expense or toxicity associated with clinical examinations. 

Imaging studies can be ordered as clinically indicated. It is not possible based upon existing data to make recommendations on the types and frequency of radiographic studies. Routine chest radiograph is indicated, while CT scans of the chest, abdomen, or head may be indicated in patients with symptoms suggestive of recurrence. When recurrence is found, full staging workup should be performed. As to date, there is no routine tumor marker, and further studies are warranted on neuron-specific enolase and chromogranin A. 

## 8. Management of Recurrences

### 8.1. Pattern of Failure

Typically MCC has a high overall incidence of distant metastases of 40%, that is, 25% at presentation, 15% after radiotherapy, as well as a 30%–65% regional recurrence rate (20% at presentation, subsequently 45%). The overall 5-year survival was 63%. It appears to be distant metastases rather than nodal relapse that account for the poor prognosis of this disease [113]. Systemic disease is nearly uniformly preceded by the appearance of nodal metastases. This suggests an orderly “cascade” pattern of spread for this tumor [118]. 

### 8.2. Treatment of Recurrence

It is vitally important that the initial treatment be optimized to reduce chance of recurrences and patients should be followed closely. Locoregional recurrence carried an ominous significance with 67% of patients subsequently dying of disease. However occasional cases of long-term disease-free survival have been reported [84]. In 1997, the Royal Marsden hospital reported that the treatment of unresectable primary or recurrent disease with radiotherapy led to valuable long-term control in four of nine patients treated. Six courses of chemotherapy were administered; one brief complete response was observed, occurring in a patient treated with cyclophosphamide, doxorubicin, and vincristine (CAV) [119]. 

The University of Texas reported that the overall survival rate for the 46 patients with recurrence was 37% [120]. Patients presenting distant disease had a median survival of 12 months, much worse than those who display local or nodal disease. For patients with nodal or local recurrence, the mean survival after combination therapy (chemotherapy, radiation ± surgery) was 36.5 months as compared with 17.5 months for those treated with a single modality (surgery or radiation or chemotherapy). Multimodality therapy has shown the best results for recurrent MCC thus far and should be used if tolerated by the patient. Aggressive salvage surgery for local or nodal recurrence is encouraged, because this disease has a tendency to become more destructive upon recurrence. Adjuvant radiotherapy should also be used, if the patient has not exceeded dose limits.

In Tai et al.'s series, successful salvage is possible with lymph node recurrence, but not for distant metastatic diseases [121]. 

## 9. Summary Recommendations and Conclusion

### 9.1. Recommended Treatment Algorithm for Different Stages

Table 9 summarizes the treatment algorithm for MCC. The basic principles are as follows. For localized disease, surgery is the standard of treatment, generally followed by adjuvant radiation therapy in most cases. SLNB is recommended because MCC metastasizes often. It aids in the selection of patients for full dissection. The node-negative patients do not need further surgical therapy. Chemotherapy, using similar regimens as small-cell carcinoma of the lung, is advised for metastatic disease. There are no data in the literature of improved survival from adjuvant chemotherapy yet, and so future research for better chemotherapeutic agents and regimens should be done. 

#### 9.1.1. Early Localized Disease

Many surgeons feel that aggressive surgical therapy is the only way to prolong survival. In other words adjuvant therapy is not able to compensate for inadequate initial surgery. If only local excision of the primary lesion was performed, local recurrence occurred in 39% and regional failure occurred in 46% [122]. Regardless of stage, patients treated with local excision alone had a 52% 5-year overall survival, while patients treated with local excision and lymph node dissection had an 87% survival rate [123]. Patients undergoing wide local excision, prophylactic lymph node dissection, and adjuvant radiotherapy had significantly decreased local/regional and distant recurrence rates and improved survival when compared with their counterparts. 

#### 9.1.2. Regional Disease

For patients who either present with regional disease or later developed regional disease, the best outcome is obtained following treatment by therapeutic lymph node dissection with or without radiotherapy postoperatively. Important studies are summarized in the following.

 Pergolizzi et al. reported a patient who had a wide excision of the primary lesion, prophylactic lymph node dissection (15 of 34 lymph nodes were positive), and adjuvant chemotherapy [124]. He was alive and disease-free at 23 months.

 Meeuwissen et al. [125] recommended wide excision of the primary site with elective postoperative radiotherapy to both the primary site, in-transit zone where practicable, and regional nodal area. If malignant nodes occur, block dissection with postoperative radiotherapy is indicated. 

 In 1995, Boyle reported that responses to chemotherapy were observed in 8 of 20 applications (40%), particularly carboplatin and etoposide given in the setting of regional node disease [126].

 Radiation therapy was highly effective when given as consolidation after surgery or chemotherapy [127, 128]: only 1/11 (9%) irradiated patients developed in-field recurrence. Radiotherapy achieved responses in 15 of 15 measurable sites (5 complete responses and 10 partial responses). Chemotherapy achieved responses in 18 of 26 patients (69%), mostly complete (41%). However, in the absence of radiotherapy, the responses were short-lived. Four of 8 patients with advanced locoregional disease were disease-free with induction chemotherapy followed by radiotherapy as consolidation regime. The chemotherapy drugs were cyclophosphamide, methotrexate, and 5-fluorouracil (CMF); 3/5 patients were disease-free at 5, 12, and 37 months. Their data support the use of combined treatment with chemotherapy followed by radiotherapy for advanced locoregional MCC.

 In conclusion, oncologists may consider adjuvant chemotherapy for patients with node-positive MCC as per NCCN guideline [9].

**(1) Nodal Presentation with no Evidence of Cutaneous Tumor**

In the presence of a nodal MCC, an exhaustive clinical search for a primary tumor must be carried out. After the exclusion of any reasonable starting point of the neoplasm, a provisional diagnosis of “primary” nodal MCC may be acceptable. Ferrara et al. reported two cases of MCC within inguinal and axillary lymph nodes, respectively, showing no clinicopathologic evidence of a primary tumor [129]. One patient was alive with no evidence of disease five years and ten months after the surgical excision of the neoplasm with no postoperative chemotherapy. In the combined data of three Australian hospitals, they had 91 patients with nodal disease at presentation, which was about 1/3 of the total cases of MCC [130]. Of these 91 patients, 40% had an occult primary. It was found that those with occult primary had a significantly better prognosis.

#### 9.1.3. Metastatic Disease

Palliative radiotherapy and chemotherapy can be given for metastatic disease. Overall response rates to combination chemotherapy for surgically unresectable distant metastatic disease are generally high, although responses are transient. Chemotherapy is considered effective in some reports [113, 125–127]. Etoposide, cisplatin, Adriamycin, and bleomycin combination chemotherapy was used in one case of metastatic MCC; complete remission was achieved which lasted 15 months [131]. Others have noted that in advanced disease, chemotherapy and radiotherapy were not able to induce long-term remission [126, 127, 132, 133]. 

The following innovative treatments show promising preliminary results.

1. Octreotide: neuroendocrine tumors have somatostatin receptors. During the past decade, these receptors have been demonstrated in vivo by octreoscan [116].
2. Lanreotide is a long-lasting somatostatin analogue with a similar binding profile to octreotide. Complete remission has been reported for disseminated MCC with a followup of 17 months [134].
3. Isolated hyperthermic limb perfusion with melphalan: an unusual mode of dissemination of this tumor is the phenomenon of in-transit metastases. Gupta et al. reported complete resolution of in-transit metastases from an MCC in response to treatment with isolated hyperthermic limb perfusion with melphalan. Limb perfusion appears to be a promising modality for such lesions [135].
4. Imiquimod combined with radiation therapy [136].
5. Pazopanib [115] and other related molecular targeted agents.

### 9.2. Treatment Outcomes

Table 10 summarizes results of treatment outcomes in large series in the literature. The most important predictor of survival was presence of lymph node metastasis (*P* = 0.03). After surgery, local recurrence occurred in 27% of patients. Lymph node metastasis was the first sign of recurrence in 60% of patients and preceded distant metastasis [137]. Recurrence at lymph node basins was lower in patients with elective lymph node dissection (0%) compared with therapeutic node dissection (57%) (*P* < 0.05). Because MCC spreads in a “cascade” fashion, elective node dissection may provide a chance for a cure. 

### 9.3. Future Research

Future areas of research include refinement of prognostic factors to select patients for adjuvant treatment, development of serum tumor markers, and more effective chemotherapy or molecular targeted agents.

There is an interesting report of four cases of stage I MCC treated with surgery followed by intralesional bleomycin. They had no evidence of recurrence or metastases on follow-up for up to five years [138]. One case was given intralesional bleomycin on an adjuvant basis. Another case had radiation postoperatively but the tumor recurred. Intralesional bleomycin produced complete regression of this tumor with minimal scarring and long-term cure. Bleomycin is an antibiotic isolated from cultures of *Streptomyces verticillus* and was found to have antineoplastic and antiviral effects on the hepatitis C virus and human immunodeficiency virus [139]. Since 1970 when it was first used for warts millions of patients have been cured by intralesional bleomycin therapy. MCC is caused by a polyoma virus, one of two genera within the Papovaviridae family. Human Papovavirus is extremely susceptible to bleomycin. Therefore, more studies should be done to confirm its efficacy.

## Figures and Tables

**Table 1:** Pathology diagnosis of MCC.

(1) Perform fine needle aspiration cytology or biopsy of different lesions in the same patient.	
(2) Section skin specimen thoroughly to avoid missing the aggressive MCC component since it can coexist with other more common skin cancers.	
(3) Immunostaining for cytokeratin-20 can detect micrometastasis in sentinel nodes.	

**Table 2:** Summary of the 2010 American Joint Committee on Cancer Merkel Cell Carcinoma staging system.

Primary tumor (T)	
Tx	Primary tumor cannot be assessed
T0	No evidence of primary tumor
Tis	In situ primary tumor
T1	Primary tumors less than or equal to 2 cm maximum tumor dimension
T2	Primary tumors greater than 2 cm but not more than 5 cm maximum tumor dimension
T3	Primary tumors over 5 cm maximum tumor dimension
T4	Primary tumor invades bone, muscle, fascia, or cartilage

Regional lymph nodes (N)	

Nx	Regional lymph nodes cannot be assessed
N0	No regional lymph node metastasis
cN0	Nodes negative by clinical exam (no pathologic node exam performed)
pN0	Nodes negative by pathologic exam
N1	Metastasis in regional lymph node(s)
N1a	Micrometastasis
N1b	Macrometastasis
N2	In-transit metastasis

Distant metastasis (M)	

M0	No distant metastasis
M1	Metastasis beyond regional lymph nodes
M1a	Metastasis to skin, subcutaneous tissues, or distant lymph nodes
M1b	Metastasis to lung
M1c	Metastasis to all other visceral sites

Anatomic stage/prognostic groups	

IA	Primary tumor ≤2 cm; regional LN negative by pathologic examination^a^
IB	Primary tumor ≤2 cm; regional LN negative by clinical examination only^b^
IIA	Primary tumor >2 cm; regional LN negative by pathologic examination^a^
IIB	Primary tumor >2 cm; regional LN negative by clinical examination only^b^
IIIA	Primary tumor any size; positive micrometastasis in regional LN^c^
IIIB	Primary tumor any size; clinically detectable regional LN metastasis and/or in-transit metastasis^d^
IV	Primary tumor any size; any distant metastasis

^a^Negative sentinel lymph node biopsy (SLNB) or elective lymph node dissection (ELND).

^b^No pathologic LN evaluation (SLNB or ELND).

^c^Positive micrometastasis by SLNB or ELND.

^d^Confirmed pathologically by biopsy or therapeutic lymph node dissection.

LN: lymph nodes.

**Table 3:** 5-year survival outcome of different stages of MCC [7].

	Percentage of all patients	5-year overall survival
Stage I and II (localized disease)	60%–70%	60%–80%
Stage III (nodal disease)	30%	50%
Stage IV (distant metastasis)	5%–10%	20%
All stages combined	100%	40%

**Table 4:** Staging workup should include the following.

(1) Complete examination of the skin (including scalp) and regional lymph nodes	
(2) Computerized tomography (CT) of chest to rule out lung metastases or small-cell carcinoma arising in the lung and metastasizing to the skin	
(3) Positron emission tomography (PET) with 18-fluorodeoxyglucose (18FDG), either alone or combined with computed tomography	

**Table 5:** Important prognostic factors for disease-free survival [8].

Favorable	
Initial localized disease	
Surgery as part of the treatment	
Adjuvant radiotherapy	
Unfavorable	
Age > 70 years	
Male sex	
Trunk site	
Head and neck site—especially lip	
Size of primary > 2 cm	
Initial nodal disease presentation, especially if >2 Involved nodes	
Initial distant disease presentation	
Histology—small-cell size, high mitotic rate, depth of invasion	
Lymphovascular invasion	
Infiltrative, rather than a nodular, growth pattern	

**Table 6:** Summary of surgical management of MCC (NCCN guidelines) [9].

(1) Surgery is the mainstay of treatment for MCC. Radiotherapy is an inferior option for cancer control since the complete response of gross disease of MCC to radiotherapy is only 75%.	
(2) It is always best to perform the SLN biopsy before definitive local excision. After wide local excision, SLN biopsy may be considered in selected patients, although accuracy of results may be compromised especially in nonextremity regions.	
(3) Resection margin: 1-2 cm. Clear surgical margins when clinically feasible but surgeon should take into account cosmetic and functional outcomes. Close or positive margins should always be followed by adjuvant radiotherapy.	
(4) Different surgical techniques: local excision, wide local excision, Mohs technique, modified Mohs (Mohs technique with additional final margin for permanent section assessment), and CCPDMA (complete circumferential and peripheral deep margin assessment).	
(5) Any reconstruction involving extensive undermining or tissue movement is delayed until negative histological margins are verified. When primary closure is not possible, consider split-thickness skin graft as it is easier to monitor recurrence.	
(6) In the head and neck region, risk of false-negative SLN biopsy is higher, due to aberrant lymph node drainage and frequent presence of multiple SLN basins. SLN biopsy is therefore not mandatory.	
(7) SLN assessment—sensitivity of cytokeratin-20 immunohistochemical staining is over 90% and must be used. It can detect micrometastasis missed by H&E staining.	

H&E: hematoxylin and eosin; NCCN: National Comprehensive Cancer Network; SLN: sentinel lymph node.

**Table 7:** Summary of important series of sentinel node biopsy (SLNB).

Institute	Number of patients	Important results/conclusions
Helsinki University Hospital, Helsinki, Finland. Koljonen et al. [10]	15 patients	(i) False-negative lymph nodes were found in 30% of the patients. Immunohistochemical reevaluation decreased this figure to 22%. Therefore for MCC, false-negative sentinel lymph nodes can and should be limited by using immunohistochemistry.

Memorial Sloan-Kettering Cancer Center, New York, United States. Fields et al. [11]	153 patients	(i) Factors associated with SLNB positivity are primary tumor size (25% ≤2 cm versus 45% >2 cm; *P* = 0.02) and presence of LVI (55% versus 4%; *P* < 0.01). The 2-year CIs of recurrence or death from MCC for LVI-positive patients were 30% and 15%, respectively. No LVI-negative patient experienced recurrence of disease or died of MCC.

Westmead Hospital, New South Wales, Australia. Howle and Veness [12]	16 patients, stage I or II	(i) 8/16 (50%) had a positive SLN. (ii) 8/16 had a negative SLN and did not undergo any nodal treatment following SLNB. Two of these patients developed nodal relapse, giving a false negative rate of 20%.

CIs: confidence intervals; LVI: lymphovascular invasion; MCC: Merkel cell carcinoma; SLNB: sentinel node biopsy.

**Table 8:** Indications for adjuvant radiotherapy; see text for more liberal indications.

(1) Primary tumor size > 2 cm	
(2) Positive resection margins or tumors that closely approximate the surgical margin	
(3) Lymphovascular invasion in the primary tumor	
(4) Extracapsular extension of tumor outside nodes	
(5) Documented regional lymph node involvement or when regional lymph nodes were not pathologically staged	

**Table 9:** Updated treatment algorithm for Merkel cell carcinoma [13–15].

Stages I and II (localized disease)	
(i) Wide local excision with SLNB. If SLN positive, complete LN dissection if feasible. If not, nodal radiotherapy	
(ii) Wide excision and prophylactic lymph node dissection	
(iii) Wide excision of the primary tumor, alone or combined with adjuvant radiotherapy	
(iv) Mohs micrographic surgery can be used if feasible	
(v) Excision followed by postoperative adjuvant radiotherapy to primary ± nodal regions	
Stage III (regional disease)	
(i) Wide local excision plus LN dissection if feasible. If not, radiation therapy to primary and nodal regions	
(ii) Adjuvant chemotherapy is controversial but may be considered in selected fit high-risk patients	
Stage IV (distant disease)	
(i) Palliative care with or without surgery, radiotherapy, and chemotherapy	

LN: lymph node; SLN: sentinel lymph node; SLNB: sentinel lymph node biopsy.

**Table 10:** Treatment outcomes in large series in the literature.

Institute	Number of patients/important features	Important results/conclusions
Memorial Sloan-Kettering Cancer Center, New York, US. Allen et al. [16]	109 retrospective	(i) Overall DSS after recurrence was 62% (ii) Predictors of improved DSS after recurrence included (a) nodal as compared to local or distant recurrence (b) the ability to render the patient free of disease after recurrence (c) DFI > 8 m

Princess Margaret Hospital, Canada; Royal Prince Alfred Hospital & Westmead Hospital, Sydney, Australia. Clark et al. [17]	110 retrospective	Predictors of survival on MVA: (i) age > 70 years (HR 6.19, *P* < 0.001), (ii) primary tumor size > 1 cm (HR 7.55, *P* < 0.001), (iii) number of nodal metastases divided into none, ≤ 2 & >2 (HR 3.71 per stratum, *P* < 0.001) (iv) stage II disease derives the greatest benefit from adjuvant RT, including improved DSS

Surveillance Epidemiology, and End Results database. Mojica et al. [18]	1665 Retrospective registry	(i) MS for those with and without adjuvant RT: 63 versus 45 m (ii) RT improves survival for patients with all sizes of tumors, particularly when primary lesions are larger than 2 cm

University of Saskatchewan. Tai et al. [19]	433 retrospective, combined with the literature cases	(i) Nodal metastases occurred clinically at presentation in 9/105 (9%) patients with primary tumor size 1 cm or less—too high to obviate SLNB even for small tumors

Peter MacCallum Cancer Centre. Hui et al. [20]	176 retrospective	(i) Median interval to recurrence was 8 m (ii) DM developed in 43 patients (24%) (iii) Age, primary tumor size, and RT (no RT versus <45 Gy versus ≥45 Gy) were predictive of locoregional control on univariate analysis However, only RT remained significant on MVA

Memorial Sloan-Kettering Cancer Center, New York, US. Fields et al. [21]	500 prospective database	(i) 50% patients died during followup: 25% died of disease, 24% died of other causes (ii) 5-year OS and CSS were 56% and 30%, respectively (iii) Only 1 of 132 patients without LVI died of MCC

University of Bern, Switzerland. Ghadjar et al. [22]	180 retrospective	(i) RT group compared to surgery to primary tumor alone: LRFS (93% versus 64%; *P* < 0.001), RRFS (76% versus 27%; *P* < 0.001), DMFS (70% versus 42%; *P* = 0.01), DFS (59% versus 4%; *P* < 0.001), CSS (65% versus 49%; *P* = 0.03) (ii) LRFS, RRFS, DMFS, and DFS are s.s. on MVA.

Helsinki University Central Hospital, Norway. Kukko et al. [5]	181 retrospective	(i) No extra benefit was gained from a wide surgical margin (≥2 cm) compared to a margin of 1–1.9 cm, but an intralesional excision was more often associated with LR

Memorial Sloan-Kettering Cancer Center, New York, US. Fields et al. [23]	364 prospective database	(i) 30% developed a recurrence: 3% local, 3% in-transit, 12% nodal, 12% distant (ii) A low recurrence rate in patients with clinically node-negative MCC was achieved with adequate surgery (including SLNB) and the selective use of adjuvant RT for high-risk tumors

Mayo Clinic, US. Grotz et al. [24]	240 retrospective	(i) 10.4% local, 7.5% in-transit, 11.3% nodal recurrences (ii) LRR is a poor prognostic sign, with a 3-year OS of 39%, but still warrants aggressive treatment

CI: confidence interval; DFI: disease-free interval; DFS: disease-free survival; DM: distant metastasis; DMFS: distant metastasis-free survival; DSS: disease-specific survival; LR: local recurrence; LRFS: local relapse-free survival; LRR: local-regional recurrence; LVI: lymphovascular invasion; m: months; MS: median survival; MVA: multivariate analysis; OS: overall survival; RRFS: regional relapse-free survival; RT: radiotherapy; SLNB: sentinel lymph node biopsy; s.s.: statistically significant; US: United States.
